# Sensitivity of *Haemonchus contortus* to anthelmintics using different in vitro screening assays: a comparative study

**DOI:** 10.1186/s13071-022-05253-3

**Published:** 2022-04-12

**Authors:** Beatriz Munguía, Jenny Saldaña, Magdalena Nieves, María Elisa Melian, Manuela Ferrer, Ramiro Teixeira, Williams Porcal, Eduardo Manta, Laura Domínguez

**Affiliations:** 1 Área de Farmacología, CIENFAR, Facultad de Química, Universidad de la República (Udelar), Montevideo, Uruguay; 2 Departamento de Química Orgánica, Facultad de Química, Universidad de la República (Udelar), Montevideo, Uruguay; 3 Departamento de Química Orgánica, Facultad de Química, Laboratorio de Química Farmacéutica, Universidad de la República (Udelar), Montevideo, Uruguay

**Keywords:** *Haemonchus contortus*, Automated motility assay, Development assay, Anthelmintic screening, Benzimidazole derivatives, New anthelmintics

## Abstract

**Background:**

Helminthiasis and resistance to commercial anthelmintic compounds are major causes of economic losses for livestock producers, resulting in an urgent need for new drugs and reliable in vitro screening tests capable of detecting potentially active products. Considering this, a series of novel benzimidazole derivatives (5-methylbenzimidazole 1,2-disubstituted, 5-carboxybenzimidazole, 5-methylbenzimidazole 2-one) was screened on exsheathed L3 (xL3) and on the adult stage of *Haemonchus contortus* (Kirby anthelmintic-susceptible McMaster isolate).

**Methods:**

This work presents the set-up of an automated motility assay on the xL3 stage of *H. contortus* using an infrared tracking device (WMicrotracker One) together with a larval development test (xL3 to L4) and a motility assay on the adult stage of *H. contortus*. A comparative study of the sensitivity of these in vitro assays using commercial anthelmintics with different mechanisms of action was carried out, also evaluating anthelmintic activity of a series of novel benzimidazole derivatives.

**Results:**

The automated xL3 assay had the great advantage of being able to analyze many compounds simultaneously, but it showed the limitation of having lower sensitivity, requiring higher concentrations of the commercial anthelmintics tested compared to those needed for the adult motility or development assays. Although none of the novel 1,2,5-tri-substituted benzimidazole derivatives could significantly decrease the motility of xL3s, one of them (1e) significantly affected the development of xL3s to L4, and five new compounds (1b, 1d, 1e, 2a and 2c) reduced the motility of *H. contortus* adult stage.

**Conclusions:**

The analysis of the results strongly suggests that the in vitro xL3 to L4 development test, particularly for the L4 stage, could be closer to the pharmacological sensitivity of the adult stage of *H. contortus* (target of interest) for commercial anthelmintic selected, with different mechanisms of action, and for the series of benzimidazole derivatives assayed. Therefore, an automated motility assay on L4 using the infrared tracking device is being set up. Further studies will be conducted to evaluate the in vivo anthelmintic activity of the most active novel benzimidazole derivatives.

**Graphical Abstract:**

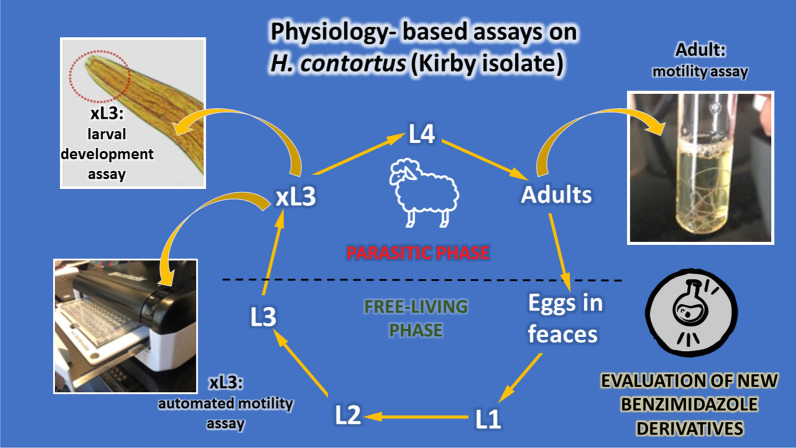

**Supplementary Information:**

The online version contains supplementary material available at 10.1186/s13071-022-05253-3.

## Background

Parasitic infections, particularly those produced by nematodes, are of interest in both human and veterinary medicine. In small ruminants, *Teladorsagia circumcincta, Haemonchus contortus* and *Trichostrongylus colubriformis* have evolved to become the main species of production-limiting parasites that affect sheep, generating significant reductions in live weight gain, wool production and fecundity [1], causing substantial economic losses [2]. For example, the annual cost associated with parasitic diseases in the Australian sheep industry for meat consumption has been estimated at 436 million USD [3], being the second largest sheep-producing country in the world. Chemical control of these parasites has been preferred because of its costs, efficacy and simplicity, but the extensive and inappropriate use of commercialized drugs has led to worldwide resistance [4–6]. The discovery and registration of new anthelmintics have been scarce in recent decades [7], with the following being the most recently introduced to the market: derquantel [8], emodepside [9] and monepantel [10]. However, reports of parasite resistance to these new drugs appeared a few years after they were marketed [11–13].

The search process for new anthelmintics has been changing in recent decades. Most of the currently commercialized anthelmintic products were discovered from the screening of compounds of natural (e.g. ivermectin [14]) or synthetic (e.g. levamisol [15]) origin, performed in infected animals. Nevertheless, the low throughput and high costs of these studies, together with the need to test a greater number of compounds, led to the development of in vitro methods involving helminths, the so-called physiology-based assays. In these tests, the parasite is cultured in vitro in the presence of the compound to be evaluated, and phenotypic parameters (viability, motility, worm growth) are studied [16–19]. These physiology-based assays still present limitations as they are not efficient enough for screening large compound libraries, in most cases because of the available subjective and manual methods to evaluate parasite response [20]. Examples of assays that include automated readings of larval motility after short exposures to different compounds are some of the breakthroughs that provide a speedy read of general anthelmintic activity. These new formats are less time-consuming and labor-intensive, although such tests cannot discriminate between death or immobile phenotype [21–23].

Haemonchosis, the infection caused by *H. contortus*, is one of the most important parasitic diseases of livestock species, affecting hundreds of millions of small ruminants and generating substantial economic losses [2]. *Haemonchus contortus* infections in young sheep and goats can persist for weeks, and each adult female worm can produce thousands of eggs per day, which can be harvested from host feces and cultured in the laboratory to obtain their infective L3 larval stage [20]. The use of the adult stage of *H. contortus* in anthelmintic activity tests requires the necropsy of the host to extract the parasites from the abomasum and the parasites immediate use for culture (they cannot be preserved). In this regard, this methodology is not feasible for performing high-throughput screenings and is reserved for secondary screening of those compounds that were active in models involving larval stages. Most recently, the use of exsheathed L3 (xL3s), the first parasitic stage of nematodes, was described for in vitro bioassays of anthelmintic activity [23]. This approach presents several advantages: the good conservation of the L3 stage parasitic material (3 months at 10 °C) without affecting phenotypes; the relatively simple exsheathment procedure of L3s; the fact that xL3s are more susceptible than L3s to the commercialized anthelmintics tested (more than 100 times) [23]; and the possibility to evaluate the success of the xL3 development to the L4 stage in vitro. Therefore, the use of xL3 and L4 is presented as an attractive option for primary in vitro screening of new anthelmintics.

In this work we present the set-up and verification of an automated motility assay for new anthelmintics discovery on xL3 stage of *H. contortus* using an infrared tracking device together with a larval development test (xL3 to L4) and a motility assay in the adult stage of *H. contortus*.

Four commercial anthelmintics, with different mechanisms of action, were evaluated in the different in vitro tests described in an effort to determine which assay has the closest sensitivity to the adult stage test (target of interest).

Finally, the anthelmintic activity of a series of previously synthetized [24], novel benzimidazole derivatives (5-methylbenzimidazole 1,2-disubstituted, 5-carboxybenzimidazole, 5-methylbenzimidazole 2-one) was evaluated.

## Methods

### Chemicals

**Fig. 1:**
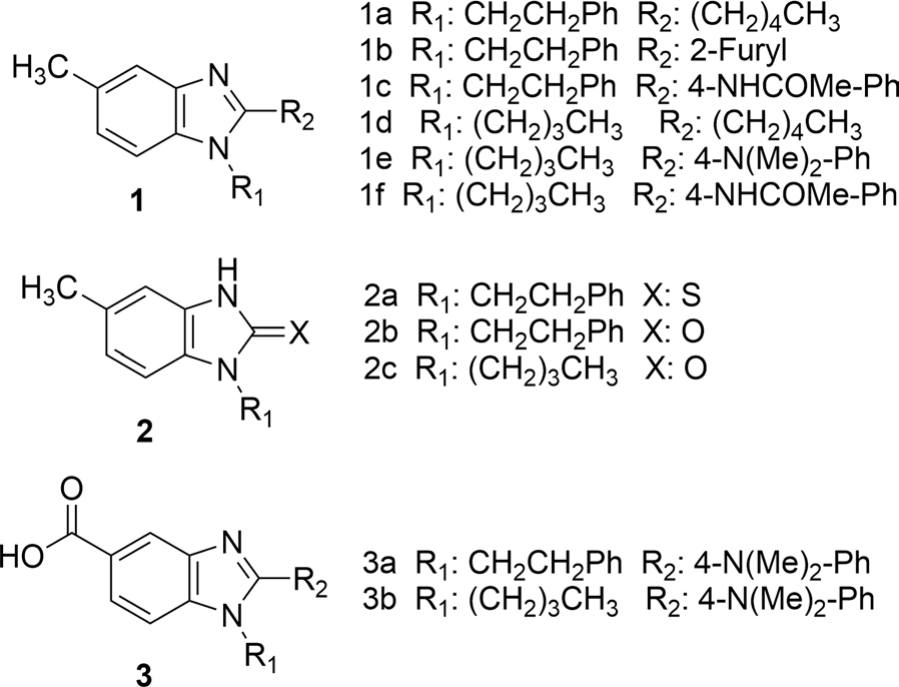
Novel benzimidazole derivatives. For chemical details, see [24]

Ivermectin (IVM), levamisole (LEV), albendazole (ABZ) and albendazole sulfoxide (ABZ SX) of USP grade were kindly supplied by Laboratorio Uruguay S.A (LUSA). Monepantel (Zolvix™) was purchased from local suppliers. A new series of 1,2,5-tri-substituted benzimidazole derivatives (chemical structures are shown in Fig. 1) was kindly supplied by Dr. Williams Porcal; the purity of the benzimidazole derivatives was ≥ 95% (assessed by 1H-NMR) [24].

### *Haemonchus contortus* production and maintenance: adult stage

Infective third-stage larvae (L3) from the anthelmintic-susceptible McMaster isolate (Kirby) were kindly provided by Dr. A. Kotze and Dr. M. Knox (CSIRO McMaster Laboratory, Armidale, NSW, Australia). The infection was maintained at the Campo Experimental del Instituto de Higiene de Facultad de Medicina, UdelaR, as we have previously described [25].

The adult worms used in this study were recovered from sheep abomasa by manual picking 25–35 days after infection with L3. The parasitic material was washed first with PBS and then with sterile supplemented RPMI culture medium (RPMI-1640 10.4 g/l—catalog number R6504, Sigma Aldrich, HEPES buffer 2.38 g/l, glucose 8 g/l, 20% of newborn bovine serum, penicillin 100 IU/ml, amphotericin B 2.5 µg/ml and streptomycin 100 µg/ml) both at 37 °C. After washing, the adult worms were incubated at 5% CO_2_ and 37 °C for 30 min before use in supplemented RPMI culture medium.

### *Haemonchus contortus* production and maintenance: exsheathed third-stage larvae (xL3s)

Third-stage larvae (L3s) were obtained from *H. contortus* eggs by incubating humidified feces from infected sheep at 27 °C for 1 week according to [26, 27]. Once collected, L3s were maintained in distilled water at 5–9 °C for up to 60 days.

L3s were cultured and exsheathed according to Preston et al. [23], with minor modifications. Briefly, L3s were exsheathed by incubation in a 0.17% w/v active Cl_2_ solution for 15 min at 40 °C and 10% CO_2_ and then washed four times in sterile 0.9% NaCl (w/v), centrifuging for 5 min at 500×*g* each time. The exsheathed L3s (xL3s) were then washed once with Luria Bertani (LB) medium supplemented with 100 IU/ml penicillin, 100 µg/ml streptomycin and 2.5 µg/ml amphotericin. Finally, the xL3s were suspended in this supplemented LB.

### In vitro anthelmintic assays: xL3 automated motility assay

The collected xL3s suspended in LB were adjusted to 6000 xL3/ml. The xL3 suspension was placed in a reservoir previously sterilized with UV radiation and homogenized by an air stream using a 0.22 µm filter membrane, and 300 xL3s (50 µl of the suspension) were transferred per well to sterile 96-well flat bottom microplates (CellStar, Greiner), with the exception of the plate edge wells, which were filled with sterile distilled water, using a multichannel pipette. Then, 50 µl of stock solutions of the compounds (prepared in LB and 1% v/v DMSO at a 2× concentration) was added to each well to obtain a final 1× concentration and 0.5% v/v DMSO (six replicates each) (see Table 1 for the drug concentration ranges assayed).

**Table 1:** Concentrations (µM) of commercial anthelmintics assayed in the different in vitro tests

	xL3s motility test	xL3s to L4 development test	Adult motility test
Albendazole sulfoxide	0.03–200	0.001–100	0.03–36
Monepantel	0.02–0.7	0.01–0.7	0.002–2
Ivermectin	0.02–2.8	0.005–2.8	0.0001–0.1
Levamisole	0.80–50	–	0.0005–5

The new benzimidazole derivatives were tested at 25 µM; the screening concentration was defined based on previous reports of xL3 motility assay [23, 28] as well as by the limitation in the solubility observed for this series of compounds in the incubation conditions. Culture medium control (50 µl LB medium), negative control (50 µl of 1% v/v DMSO LB, giving a final concentration of 0.5% v/v DMSO in the well) and positive control (50 µl of albendazole 40 µM in 1% v/v DMSO LB, giving a final concentration of albendazole 20 µM and 0.5% v/v DMSO in the well) were also placed in each plate. The plates were incubated at 40 °C in a humidified incubator with a 10% CO_2_ atmosphere for 72 h. The motility assessment of the xL3 stage was performed using an automated tracking apparatus: WMicrotracker One (Phylumtech, Argentina). Briefly, worm motility measurements of each well were carried out at 24, 48 and 72 h for 30 min at 20–22 °C. Before analyzing, the plates were agitated in an orbital shaker at 180 rpm for 6 min to stimulate the worms, corroborating the normal sinusoidal movement of the xL3s in the culture medium control wells under the microscope. The WMicrotracker One uses an infrared tracking device which detects animal movement through infrared microbeam light scattering where each well is crossed by two infrared microbeams that scan more than ten times per second and are interrupted when worms pass by. The detected signal is digitally processed to calculate the amount of animal movement in a fixed period of time (http://www.phylumtech.com/home/es/soporte) [22, 29]. The raw motility data were normalized against the motility measurements obtained for the DMSO control group to remove plate-to-plate variation by calculating the percentage of motility of each treatment as follows.

1 $$\%\, {\text{of motility}}=\frac{\text{motility of treatment}}{\text{motility of DMSO control}}*100.$$

For assay verification, the data from the concentration-response curves of the different commercial anthelmintics (used as reference drugs) were transformed to log10 to determine the concentration that leads to 50% maximal response (IC_50_) and 10% maximal response (IC_90_) from three independent trials. The data were adjusted using a variable slope and a four-parameter logarithmic equation (Eq. 2), where A1 is the bottom asymptote, A2 is the top asymptote, log x_0_ is the center of the curve and represents the log IC_50_, and p is the Hill variable slope of the logarithmic dose-response curve.

2 $$y=A1+\frac{A2-A1}{1+{10}^{\left({logx}_{0 }-x\right)*p}}$$

The IC_50_ (Eq. 3) and IC_90_ (Eq. 4) were determined using GraphPad Prism version 9.0.2, 2021, for Windows (GraphPad software, San Diego, CA, USA).

3 $${\mathrm{IC}}_{50}= {10}^{{logx}_{0}}$$

4 $${\mathrm{IC}}_{90}= {10}^{{(logx}_{0}+log0.11)*p}$$

To study whether the vehicle used, DMSO, affects the motility of the larvae at 24, 48 and 72 h of incubation, we compared the motility of the medium control with the DMSO control for each experiment. We applied an unpaired t-test with Welch correction and a Holm-Šídák multiple comparisons test, using GraphPad Prism version 9.0.2, 2021, for Windows (GraphPad software, San Diego, CA, USA). A probability of *P* < 0.05 was considered statistically significant.

### In vitro anthelmintic assays: xL3 to L4 development assay

For the xL3 to L4 development assay, we proceeded according to bibliography [23] with minor modifications. In brief, the plates tested for xL3 motility were re-incubated for 4 more days at 40 °C and 10% v/v CO_2_, completing a total of 7 days of incubation. Then, worms were fixed with 50 µl of a 1% iodine solution, and 20 µl of each well was examined at 10× magnification (Nikon TS100, inverted microscope, Tokyo, Japan) to assess their development, based on the presence or absence of a well-developed pharynx characteristic of *H. contortus* L4s (see Additional file 1: Fig. S1). The number of L4s was expressed as a percentage of the total worm number present in the 20 µl larval suspension examined under the microscope. Each concentration of anthelmintics or the new benzimidazole derivatives was tested in triplicate, at least in three different trials. Also, to study whether the vehicle used, DMSO, affects the development of the xL3 to L4 larvae at 7 days of incubation, we compared the development of the medium control with the DMSO control for each experiment. We applied an unpaired t-test with Welch correction and a Holm-Šídák multiple comparisons test, using GraphPad Prism version 9.0.2, 2021, for Windows (GraphPad software, San Diego, CA, USA). A probability of *P* < 0.05 was considered statistically significant.

### In vitro anthelmintic assays: adult stage motility assay

This assay was carried out according to O’Grady and Kotze [19], with minor modifications. Briefly, ten adult worms were placed into glass tubes containing 2 ml of sterile supplemented RPMI culture medium. Stock solutions of the compounds in 100% DMSO were prepared at a 200× concentration to obtain final drug concentrations of 1× and 0.5% DMSO in each tube when 10 µl of stock solutions was incorporated (six replicates at each drug concentration tested, see Table 1). Culture medium and 0.5% DMSO in culture medium controls were also included in each assay. The new benzimidazole derivatives were tested at a final concentration of 25 µM (six replicates). The worms in the tubes were incubated at 37 °C and 5% CO_2_ for 72 h, and the motility score was determined at 0, 24, 48 and 72 h according to the following scoring system: 0 = no movement, 1 = very limited movement and not in a normal sinusoidal form; 2 = at least one individual is able to move in a normal sinusoidal form; 3 ≥ 50% of the individuals present smooth sinusoidal motion. A compound with a motility score ≤ 1.5 at 72 h was considered active. Each drug assay was carried on three separate occasions.

### Statistical analysis

An ANOVA test was conducted in each assay (once the normal distribution of data was confirmed) followed by Dunnett’s multiple comparison test against the DMSO control group, with 95% confidence using GraphPad Prism version 9.0.2, 2021, for Windows (GraphPad software, San Diego, CA, USA). A probability of *P* < 0.05 was considered statistically significant. The Dunnett’s multiple comparison test is restricted and recommended for its use comparing several experimental groups against a single control group [30], as the DMSO control.

## Results

### Set-up and verification of anthelmintic in vitro assays using *H. contortus* (xL3s, xL3 to L4 development and adults)

The xL3 automated motility assay and xL3 to L4 development assay were verified against four commercial anthelmintics with different mechanisms of action: albendazole sulfoxide, monepantel, ivermectin and levamisole [31], at the range of concentrations indicated in Table 1. The IC_50_ and IC_90_ values were determined for these compounds at 24, 48 and 72 h of incubation for the xL3 motility assay and at day 7 of incubation for the xL3 to L4 development assay (Table 2 for IC_50_ values and Additional file 1: Table S1 for IC_90_ values). The log_10_ concentration-response curves are provided as supplementary data (Additional file 1: Figs. S2–S8). Also, the influence of DMSO on the motility and development of the xL3 larvae was studied, showing no significant differences (unpaired t-test with Welch correction and a multiple comparison Holm-Šídák post test, *P* < 0.05), with the motility or development of xL3s of the medium control group, in each experiment carried out (Additional file 1: Tables S2–S5).

**Table 2:** Half of the maximum inhibitory concentrations (IC_50_) for commercial anthelmintics evaluated in the *Haemonchus contortus* in vitro assays: exsheathed third-stage (xL3) automated motility assay and xL3 development to fourth stage (L4)

Incubation time	xL3 motility assay IC_50_ (mean ± SD) µM			xL3 to L4 development assay IC_50_ (mean ± SD) µM
	24 h	48 h	72 h	7 days
Albendazole sulfoxide	NF	2.460 ± 2.370	2.067 ± 0.653	0.464 ± 0.140
Monepantel	0.145 ± 0.022	0.114 ± 0.003	0.068 ± 0.001	0.048 ± 0.013
Ivermectin	0.162 ± 0.048	NF	0.771 ± 0.087	0.077 ± 0.028
Levamisole	5.392 ± 0.611	6.053 ± 0.384	7.672 ± 0.506	ND

NF: IC_50_ value could not be determined because of curve fitting error

ND: value was not determined because of difficulties in characterizing the morphology of drug-affected larvae

The adult stage motility assay was optimized and verified against albendazole sulfoxide, monepantel, ivermectin and levamisole. The motility of worms in culture medium (culture medium control) or vehicle control (negative control) showed a mean motility score of 2.8 ± 0.3 and 2.6 ± 0.4, respectively, at 72 h, thus showing a normal sinusoidal movement during the cultivation time. The mean motility scores achieved at 72 h of incubation with anthelmintic drugs, for four different concentrations, are presented in Table 3. The effects of these anthelmintics on the motility of *H. contortus* adult stage at 24, 48 and 72 h are presented in the Additional file 1: Figs. S9–S12.

**Table 3:** Commercial anthelmintic drugs and their activity at different concentrations for each *Haemonchus contortus* assay

Drug concentration (µM)		% of motility on xL3s (average ± SEM)^a^	% of L4 development (average ± SEM)^b^	Adult stage motility score (average ± SEM)^a^
Ivermectin	1	**25.8 ± 11.6**^**c**^	**1.4 ± 2.7**^**d**^	**0.7 ± 0.5**^**e**^
	0.1	106.0 ± 21.7	**37.0 ± 9.5**^**d**^	**0.6 ± 0.2**^**e**^
	0.01	104.9 ± 23.5	66.9 ± 20.7	**0.7 ± 0.4**^**e**^
	0.001	NT	86.7 ± 21.4	**0.8 ± 0.3**^**e**^
	0.0001	NT	NT	**1.3 ± 0.1**^**e**^
Levamisole	50	**0.6 ± 1.6**^**c**^	**0.0 ± 0.0**^**d**^	**NT**
	5	72.6 ± 13.4	**8.9 ± 3.7**^**d**^	**0.0 ± 0.0**^**e**^
	0.5	128.1 ± 25.6	89.5 ± 3.1	**0.0 ± 0.0**^**e**^
	0.005	NT	NT	1.9 ± 0.2
	0.0005	NT	NT	2.8 ± 0.1
Monepantel	2	**3.5 ± 3.7**^**c**^	**4.0 ± 1.1**^**d**^	**0.3 ± 0.2**^**e**^
	0.2	**9.3 ± 7.1**^**c**^	**20.1 ± 18.0**^**d**^	**0.7 ± 0.2**^**e**^
	0.02	127.2 ± 26.3	85.5 ± 20.5	**1.0 ± 0.2**^**e**^
	0.002	NT	NT	2.2 ± 0.1
Albendazole sulfoxide	36	**37.5 ± 14.5**^**c**^	**0.0 ± 0.0**^**d**^	**1.0 ± 0.0**^**e**^
	3.6	**56.4 ± 13.5**^**c**^	**1.9 ± 2.1**^**d**^	**1.4 ± 0.3**^**e**^
	0.36	134.8 ± 27.5	**55.1 ± 16.9**^**d**^	**1.3 ± 0.2**^**e**^
	0.036	116.2 ± 20.2	74.5 ± 15.4	2.3 ± 0.0

Bold numbers indicate that the drug was active at the concentration tested

^a^Measured at 72 h of drug exposure

^b^Measured at 7 days of drug exposure

*NT* not tested

^c^Motility was significantly different from motility of DMSO control (*P* < 0.05) (see “Statistical analysis” section for further details)

^d^Development was significantly different from development of DMSO control (*P* < 0.05)) (see “Statistical analysis” section for further details)

^e^Motility score of a compound at 72 h time point ≤ 1.5 is considered active[19]

To analyze the differences in the range of concentrations at which anthelmintics are active in the three assays against *H. contortus*, the results obtained for four different concentrations of anthelmintic drugs in the xL3 motility assay, the xL3 to L4 development assay and the adult stage motility test are presented in Table 3. Additionally, Table S6 of the supplementary data section (Additional file 1) shows these results informed with their corresponding coefficient of variation.

### Screening of new benzimidazole derivatives against different stages of *H. contortus*

A series of new benzimidazole derivatives were tested at 25 µM against xL3s (xL3 automated motility assay and xL3 to L4 development assay) and the adult stage (adult motility assay). The results of these three assays are shown in Table 4 (Additional file 1: Table S7 shows these results informed with their corresponding coefficient of variation). Albendazole was tested at 20 µM as a positive control.

**Table 4:** Activity of a novel series of benzimidazole derivatives (at 25 µM) for each *Haemonchus contortus* assay (incubation time of 72 h for xL3 or adult stage motility test and 7 days for L4 development test)

Compound ID^a^	% of motility on xL3s (average ± SEM)	% of L4 development (average ± SEM)	Adult stage motility score (average ± SEM)
1a	93.1 ± 30.1	67.2 ± 4.9	2.2 ± 0.4
1b	131 ± 33.5	103.8 ± 9.6	**1.5 ± 0.5**^**d**^
1c	144.8 ± 39.5	106.1 ± 9.4	2.0 ± 0.0
1d	177.6 ± 56.1^b^	68.2 ± 18.4	**0.1 ± 0.1**^**d**^
1e	179.6 ± 49.9^b^	**30.9 ± 15.1**^**c**^	**0.1 ± 0.1**^**d**^
1f	113.8 ± 25.7	101.9 ± 5.4	2.0 ± 0.0
2a	193.1 ± 51.2^b^	111.2 ± 9.1	**1.2 ± 1.1**^**d**^
2b	131.0 ± 34.9	108.9 ± 6.7	2.3 ± 0.5
2c	134.5 ± 33.9	101.4 ± 13.6	**1.4 ± 0.5**^**d**^
3a	193.3 ± 32.3^b^	105.5 ± 7.4	2.2 ± 0.4
3b	156.7 ± 18.1^b^	100.0 ± 8.3	2.2 ± 0.4
ABZ ^e^	**29.4 ± 11.1**^**b**^	**0.6 ± 1.2**^**c**^	**1.0 ± 0.3**^**d**^

Bold numbers indicate that the compound was considered active

^a^The chemical synthesis and structure of the compounds was previously reported by [24]

^b^Motility was significantly different from motility of DMSO control (*P* < 0.05) (see “Statistical analysis” section for further details)

^c^Development was significantly different from development of DMSO control (*P* < 0.05)) (see “Statistical analysis” section for further details)

^d^Motility score of a compound at 72 h time point ≤ 1.5 is considered active[19]

^e^ABZ was evaluated at a concentration of 20 µM

## Discussion

Parasitic helminth infections are a serious health problem which, added to the resistance phenomena to most commercial anthelmintics, results in great economic losses for livestock production worldwide [32, 33]. In this context, it is necessary to invest in the search for new anthelmintics and to have validated screening bioassays for their evaluation.

Several physiology-based screening strategies using larval stages of parasitic nematodes have been developed, where the phenotypes of motility (larval motility assay, LMA) [34, 35] and development (larval development assay, LDA) [36, 37] were the most studied. The LDA is an assay that facilitates the detection of molecules that could present fast or slow mechanisms of action, since activity is read out after 7 days of incubation. For instance, the LDA allows to detect active molecules such as benzimidazole anthelmintics (with a slow mechanism of action, involving the inhibition of microtubule polymerization) but also macrocyclic lactones (that present a faster mechanism by affecting the neuromuscular system of the parasite). On the other hand, the LMA is often more sensitive to compounds with fast mechanisms of action, since the activity readings are performed at 24 h of incubation. In both tests, LDA and LMA, the phenotypes are measured by visual inspection under the microscope, and although they have been very useful for the discovery of new hit compounds for anthelmintic development, they are time-consuming and subjective and involve the use of free-living parasite stages instead of the parasitic stages of greatest interest as the pharmacological target. To deal with these problems, automatic motility reading methods have been developed, such as the micromotility meter [21, 38], designed to measure the motility of nematodes in culture tubes. More recently, automated reading methods were designed for multiwell plates, measuring motility of nematodes through electrical interference [39] or video acquisition and analysis [23, 40], allowing screening of a greater number of compounds. The measurement of the motility phenotype is a rapid detection method in order to study the affectation of the normal physiology of the parasite, which generally ends up affecting their viability [41]. On the other hand, bioassays where the viability or death of the parasite is measured as a phenotype require the use of chemical reactions that indicate an affectation of mitochondrial metabolism, making it difficult to process big libraries of chemical compounds [42, 43].

In this work, an automated motility assay using the first parasitic stage of *H. contortus*, the xL3s, was successfully set up with the WMicrotracker One equipment. In fact, the xL3 automated motility assay was verified using four commercial anthelmintics with different mechanisms of action (monepantel, albendazole sulfoxide, levamisole and ivermectin).

The IC_50_ value obtained for monepantel was of the same order of concentration as that previously reported for motility assays in xL3s at 72 h of incubation [28, 44, 45]. Interestingly, the IC_50_ values obtained for levamisole and ivermectin at 72 h of incubation were significantly higher than the IC_50_ values reached after 24 h of incubation (unpaired *t* test with Welch correction, for levamisol *t* = 4.962, *df* = 3.876, *P* = 0.0083 and for ivermectin *t* = 10.630, *df* = 3.114, *P* = 0.0015), showing a decrease in activity with incubation time without total reversion (Table 2). In this sense, some studies have shown that the nematode ATP binding cassette (ABC) transporter proteins such as P-glycoproteins (P-gp) or multidrug resistance proteins (MDR) are involved in avermectin and levamisole detoxification and in resistance development [31, 46]. In addition, studies of the transcription patterns of ABC transporters in susceptible (Kirby) and resistant (Wallangra) isolates of *H. contortus* L3 stage, after 3 h of in vitro exposure to ivermectin or levamisole, only showed an increase of the transcription of multiple ABC transporter genes in the resistant isolate [47]. It would be possible that the transcriptional response observed at 3 h in resistant L3 is delayed in susceptible L3 [47]; therefore, longer incubation times with these drugs could lead to a decrease in sensitivity, such as that we observed at 72 h of incubation in the xL3 motility assay. Contrarily, moxidectin, a derivative of milbemycin with low affinity for P-gp transporters [48, 49], did not show a decrease in its activity with incubation time in an automated xL3 motility test [28].

In addition to the automated xL3 motility assay, an xL3 to L4 development assay was set up and verified as well as a motility test against the adult stage of *H. contortus*. In the adult stage motility assay, motility scores obtained in this work for albendazole, ivermectin and levamisole were the same as those reported by O’Grady et al. [19], at the same concentrations and incubation times tested.

The use of the adult stage for drug screening is of great interest since it ensures that the drug is effective against the parasite life stage to be targeted in vivo. The IC_50_ values obtained in the xL3 motility assay were 1.4, 4.5 and 10 times greater than the IC_50_ values obtained in the xL3 to L4 development assay for monepantel, albendazole sulfoxide and ivermectin, respectively (Table 2). These results were consistent with the sensitivity observed for the LDA versus the LMA. Although the LDAs had relatively prolonged times to read out, they proved to be much more sensitive to standard anthelmintics than a 24-h test that directly measured the effects of anthelmintics on L3 motility [7].

The results obtained with the adult motility assay showed that lower concentrations of anthelmintics were necessary to affect adult motility compared to those needed to affect xL3 motility (Table 3). This observation was particularly notable for ivermectin, which affected adult motility over the entire range of concentrations tested (Table 3).

These differences in the pharmacological sensitivity to drugs observed for the different stages of *H. contortus* could be explained in terms of distinct parasitic drug bioavailabilities (parasitic diffusion and biotransformation of xenobiotics) [50] or dissimilar drug target expressions [51]. In particular, it has been reported that the expression of most cytochrome P-450 enzymes (CYP450, phase I biotransformation) in *H. contortus* was higher in the larval stages L1 and L3 than in the adult stage [52]; therefore, the differential expression of these enzymes could lead to differences in drug sensitivity between larval and adult stages [53].

Finally, assays using different stages of *H. contortus* were utilized to evaluate the anthelmintic activity of a new series of benzimidazole derivatives synthesized [24]. The benzimidazole ring is a very important and versatile pharmacophore in drug discovery, and its derivatives are an important class of bioactive molecules in the pharmaceutical field [54, 55]. The differences observed for the measured activity of these compounds in the three assays follow the same behavior observed for the commercial drugs: the sensitivity of the adult stage motility assay is higher than those of the development to L4 and xL3 motility assays, the xL3 motility assay being the least sensitive. This observation could also be related to the different drug bioavailability levels reaching each parasite stage. Although none of these new compounds could significantly decrease the motility of xL3s, one of them (1e) significantly affected the development of xL3 to L4 (ANOVA, *F*_(11,24)_ = 7.399 and *P* < 0.0001, followed by Dunnett’s multiple comparison test, *P* = 0.0025), and five new compounds (1b, 1d, 1e, 2a and 2c) affected the motility of *H. contortus* adult stage. The most remarkable results regarding these anthelmintic activity screening were obtained with compounds 1d and 1e, which affected adult motility even more than the positive control albendazole.

The effects of structural modifications to benzimidazole-like molecules on microtubule inhibitory activity have been reviewed by Lacey [56]. This author has postulated that the presence of a carbamate group in the 2 position is essential for potent microtubule inhibitory activity and alkylation of the -*N*- at the 1 position of the benzimidazole ring reduces activity 10- to 20-fold, regardless of the size of the substituent in the 5 position. Another important observation is that the pharmacological effect depends heavily on the nature of the molecule adjacent to the benzimidazole ring system [57, 58]. The new compounds evaluated in the present work are 1,2,5-tri-substituted benzimidazole derivatives, which would not meet the structure–activity relationships conditions necessary to bind to tubulins at the anthelmintic benzimidazole binding site. The antifungal benomyl, which is a 1,2-di-substituted benzimidazole, binds with low affinity to nematode β-tubulin [59] and mammalian tubulin [60] to a different site from the well-characterized colchicine and vinblastine binding site [61]. These new benzimidazole derivatives, like benomyl, could bind tubulins at a different site from 2-methyl-benzimidazole carbamate, or present a different drug target, so further studies should be conducted to identify the pharmacological target of these potential anthelmintic compounds.

## Conclusions

We successfully set-up an automated motility assay for xL3s of *H. contortus* using the WMicrotracker One equipment, complemented with an xL3 to L4 development assay and a motility test in adult worms. The automated assay presented the advantage of being able to analyze a large number of compounds, but has the limitation of having lower sensitivity to the commercial anthelmintics tested since lower concentrations of anthelmintics were necessary to affect adult motility or xL3 to L4 development compared to those needed to affect xL3 motility. The analysis of the results led to interest in the set-up and verification of an automated motility assay with the WMicrotracker using the L4 stage of *H. contortus*. In addition, we could explore the influence of inhibitors of the xenobiotic metabolism enzymes and efflux transporter proteins on the sensitivity of the xL3 motility assay. A deeper knowledge of parasite pharmacokinetics can lead to a better understanding of the different drug sensitivities observed among the parasitic stages. This information could guide the design of more reliable in vitro models to test anthelmintic activity using a larval stage that closely resembles adult worms, as could be the case in the L4 stage. Finally, one new 1,2,5-tri-substituted benzimidazole derivative (1e) significantly (ANOVA followed by Dunnett’s multiple comparison test, *F*_(11,24)_ = 7.399, *P* = 0.0025) affected the development of xL3 to L4, and five derivatives (1b, 1d, 1e, 2a and 2c) presented a motility score ≤ 1.5 in the motility test against the adult stage, classifying them as active. Particularly, compounds 1d and 1e affected adult motility even more than the positive control albendazole. These results encourage us to go deeper into the biological characterization of these active compounds and evaluate their in vivo activity in sheep infected with *H. contortus*.

## Supplementary Information

**Additional file 1: Table S1**. The present table shows the IC_90_ values obtained from the set-up and verification of in vitro assays in *H. contortus*: exsheathed third-stage (xL3) automated motility assay and xL3 development to fourth-stage (L4). **Table S2**. xL3 motility in the medium and DMSO control groups at 24 h of incubation. Statistical analysis and study of the existence of significant differences between the medium control group and DMSO control group for each experiment. **Table S3.** xL3 motility in the medium and DMSO control groups at 48 h of incubation. Statistical analysis and study of the existence of significant differences between the medium control group and the DMSO control group for each experiment. **Table S4.** xL3 motility in the medium and DMSO control groups at 72 h of incubation. Statistical analysis and study of the existence of significant differences between the medium control group and the DMSO control group for each experiment. **Table S5.** xL3 % of development in the medium and DMSO control groups at 7 days of incubation. Statistical analysis and study of the existence of significant differences between the medium control group and the DMSO control group for each experiment. **Table S6.** Commercial anthelmintic drugs and their activity at different concentrations for each *H. contortus* assay. Bold numbers indicate that the drug was active at the concentration tested. **Table S7.** Activity of a novel series of benzimidazole derivatives (at 25 µM) for each *H. contortus* assay (incubation time of 72 h for xL3 or adult stage motility test and 7 days for L4 development test). Bold numbers indicate that the compound was considered active. **Figure S1**. The present figure shows the morphological differences between xL3 and L4 stages of *H. contortus*, where the most notable distinction is the well-developed pharynx and the presence of a complete buccal capsule in L4 stage. **Figure S2.** Albendazole sulfoxide dose-response curve in xL3 automated motility assay at 24 h (dotted line), 48 h (dashed line) and 72 h (solid line). Each data point represents mean ± SEM of three different experiments with six replicates for each concentration. **Figure S3.** Monepantel dose-response curve in xL3 automated motility assay at 24 h (dotted line), 48 h (dashed line) and 72 h (solid line). Each data point represents mean ± SEM of three different experiments, with six replicates for each concentration. **Figure S4.** Ivermectin dose-response curve in xL3 automated motility assay at 24 h (dotted line), 48 h (dashed line) and 72 h (solid line). Each data point represents mean ± SEM of three different experiments with six replicates for each concentration. **Figure S5**. Levamisole dose-response curve in xL3 automated motility assay at 24 h (dotted line), 48 h (dashed line) and 72 h (solid line). Each data point represents mean ± SEM of three different experiments with six replicates for each concentration. **Figure S6.** Albendazole sulfoxide dose-response curve in xL3 to L4 development assay. Each data point represents mean ± SEM, of three different experiments, with three replicates for each concentration. **Figure S7.** Monepantel dose-response curve in xL3 to L4 development assay. Each data point represents mean ± SEM of three different experiments with three replicates for each concentration. **Figure S8.** Ivermectin dose-response curve in xL3 to L4 development assay. Each data point represents mean ± SEM of three different experiments with three replicates for each concentration. **Figure S9.** Albendazole sulfoxide effect on motility of *H. contortus* adult stage at 24 h (dotted line), 48 h (dashed line) and 72 h (solid line). Each data point represents mean ± SEM motility score of three different experiments with six replicates for each concentration. Red dotted line indicates a motility score of 1.5, a value at which a product is considered active. **Figure S10**. Monepantel effect on motility of *H. contortus* adult stage at 24 h (dotted line), 48 h (dashed line) and 72 h (solid line). Each data point represents mean ± SEM motility score of three different experiments with six replicates for each concentration. Red dotted line indicates a motility score of 1.5, a value at which a product is considered active. **Figure S11.** Ivermectin effect on motility of *H. contortus* adult stage at 24 h (dotted line), 48 h (dashed line) and 72 h (solid line). Each data point represents mean ± SEM motility score of three different experiments with six replicates for each concentration. Red dotted line indicates a motility score of 1.5, a value at which a product is considered active. **Figure S12**. Levamisole's effect on motility of *H. contortus* adult stage at 24 h (dotted line), 48 h (dashed line) and 72 h (solid line). Each data point represents mean ± SEM motility score of three different experiments with six replicates for each concentration. Red dotted line indicates a motility score of 1.5, a value at which a product is considered active.

## Data Availability

The datasets during and/or analyzed during the current study available from the corresponding author on reasonable request.
