# Supplementary material for: Sensitivity of Haemonchus contortus to anthelmintics using different in vitro screening assays: a comparative study
Source: Parasit Vectors. 2022 Apr 12;15:129. doi: 10.1186/s13071-022-05253-3 (PMC9006605; doi:10.1186/s13071-022-05253-3)
Supplement: Supplementary file 1 — Additional file 1: Table S1. The present table shows the IC90 values obtained from the set-up and verification of in vitro assays in H. contortus: exsheathed third-stage (xL3) automated motility assay and xL3 development to fourth-stage (L4). Table S2. xL3 motility in the medium and DMSO control groups at 24 h of incubation. Statistical analysis and study of the existence of significant differences between the medium control group and DMSO control group for each experiment. Table S3. xL3 motility in the medium and DMSO control groups at 48 h of incubation. Statistical analysis and study of the existence of significant differences between the medium control group and the DMSO control group for each experiment. Table S4. xL3 motility in the medium and DMSO control groups at 72 h of incubation. Statistical analysis and study of the existence of significant differences between the medium control group and the DMSO control group for each experiment. Table S5. xL3 % of development in the medium and DMSO control groups at 7 days of incubation. Statistical analysis and study of the existence of significant differences between the medium control group and the DMSO control group for each experiment. Table S6. Commercial anthelmintic drugs and their activity at different concentrations for each H. contortus assay. Bold numbers indicate that the drug was active at the concentration tested. Table S7. Activity of a novel series of benzimidazole derivatives (at 25 µM) for each H. contortus assay (incubation time of 72 h for xL3 or adult stage motility test and 7 days for L4 development test). Bold numbers indicate that the compound was considered active. Figure S1. The present figure shows the morphological differences between xL3 and L4 stages of H. contortus, where the most notable distinction is the well-developed pharynx and the presence of a complete buccal capsule in L4 stage. Figure S2. Albendazole sulfoxide dose-response curve in xL3 automated motility assay at 24 h (dotte [file 13071_2022_5253_MOESM1_ESM.docx]

**Additional file 1**

**Table S1-** The present table shows the IC_90_ values obtained from the set-up and verification of *in vitro* assays in *Haemonchus contortus*: exsheathed third-stage (xL3) automated motility assay and xL3 development to fourth-stage (L4).

|  | xL3s motility assay  IC_90_ (mean ± SD) µM | | | xL3 to L4 development assay  IC_90_ (mean ± SD) µM |
| --- | --- | --- | --- | --- |
| Incubation time | 24 h | 48 h | 72 h | 7 days |
| Albendazole Sulfoxide | NF | 23.271 ± 42.932 | 14.216 ± 7.707 | 0.785 ± 0.140 |
| Monepantel | 0.474 ± 0.185 | 0.199 ± 0.002 | 0.136 ± 0.003 | 0.288 ± 0.073 |
| Ivermectin | 0.536 ± 0.203 | NF | 1.333 ± 0.399 | 0.868 ± 0.363 |
| Levamisole | 15.994 ± 2.475 | 18.576 ± 1.169 | 22.191 ± 1.646 | ND |

NF: IC_90_ value could not be determined due to curve fitting error

ND: value was not determined due to difficulties in characterizing the morphology of drug affected larvae

**Table S2**- xL3s motility in the medium and DMSO control groups, at 24 h of incubation. Statistical analysis and study of the existence of significant differences between the medium control group and the DMSO control group for each experiment.

| Incubation time | 24 h | | Unpaired t test with Welch correction and a multiple comparison Holm-Šídák with a *P* < 0.05 | | | | | | | | |
| --- | --- | --- | --- | --- | --- | --- | --- | --- | --- | --- | --- |
| Experiment | Motility of medium control (mean ± SD) | Motility of DMSO control (mean ± SD) | Below threshold? | P value | Mean of medium control | Mean of DMSO control | Difference | SE of difference | t ratio | df | Adjusted P Value |
| 1 | 64 ± 18 | 58 ± 18 | No | 0.57647 | 64 | 58 | 6 | 10.39 | 0.5774 | 10 | 0.994228 |
| 2 | 62 ± 18 | 61 ± 22 | No | 0.933096 | 62 | 61 | 1 | 11.6 | 0.08617 | 9.623 | 0.999035 |
| 3 | 62 ± 12 | 63 ± 16 | No | 0.905137 | 62 | 63 | -1 | 8.165 | 0.1225 | 9.273 | 0.999035 |
| 4 | 110 ± 20 | 99 ± 12 | No | 0.280597 | 110 | 99 | 11 | 9.522 | 1.155 | 8.187 | 0.948388 |
| 5 | 126 ± 12 | 125 ± 15 | No | 0.90118 | 126 | 125 | 1 | 7.842 | 0.1275 | 9.54 | 0.999035 |
| 6 | 117 ± 30 | 98 ± 22 | No | 0.241901 | 117 | 98 | 19 | 15.19 | 1.251 | 9.171 | 0.937301 |
| 7 | 101 ± 9 | 98 ± 10 | No | 0.597027 | 101 | 98 | 3 | 5.492 | 0.5462 | 9.891 | 0.994228 |
| 8 | 108 ± 14 | 102 ± 12 | No | 0.444355 | 108 | 102 | 6 | 7.528 | 0.7971 | 9.771 | 0.983648 |
| 9 | 107 ± 18 | 110 ± 19 | No | 0.784626 | 107 | 110 | -3 | 10.68 | 0.2808 | 9.971 | 0.997848 |
| 10 | 98 ± 20 | 90 ± 7 | No | 0.389614 | 98 | 90 | 8 | 8.651 | 0.9248 | 6.207 | 0.980732 |
| 11 | 102 ± 25 | 85 ± 10 | No | 0.168743 | 102 | 85 | 17 | 10.99 | 1.547 | 6.56 | 0.869055 |
| n= 6 in each experiment | |  |  |  |  |  |  |  |  |  |  |

Table S3- xL3s motility in the medium and DMSO control groups, at 48 h of incubation. Statistical analysis and study of the existence of significant differences between the medium control group and the DMSO control group for each experiment.

| Incubation time | 48 h | | Unpaired t test with Welch correction and a multiple comparison Holm-Šídák post test, with a *P* < 0.05 | | | | | | | | |
| --- | --- | --- | --- | --- | --- | --- | --- | --- | --- | --- | --- |
| Experiment | Motility of medium control (mean ± SD) | Motility of DMSO control (mean ± SD) | Below threshold? | P value | Mean of medium control | Mean of DMSO control | Difference | SE of difference | t ratio | df | Adjusted P Value |
| 1 | 64 ± 14 | 58 ± 15 | No | 0.490281 | 64 | 58 | 6 | 8.377 | 0.7163 | 9.953 | 0.982462 |
| 2 | 46 ± 16 | 39 ± 9 | No | 0.378037 | 46 | 39 | 7 | 7.494 | 0.934 | 7.876 | 0.973136 |
| 3 | 49 ± 8 | 48 ± 16 | No | 0.89476 | 49 | 48 | 1 | 7.303 | 0.1369 | 7.353 | 0.988925 |
| 4 | 77 ± 37 | 85 ± 13 | No | 0.634497 | 77 | 85 | -8 | 16.01 | 0.4997 | 6.216 | 0.982462 |
| 5 | 90 ± 19 | 99 ± 13 | No | 0.363724 | 90 | 99 | -9 | 9.399 | 0.9576 | 8.84 | 0.973136 |
| 6 | 106 ± 22 | 94 ± 17 | No | 0.316817 | 106 | 94 | 12 | 11.35 | 1.057 | 9.402 | 0.967578 |
| 7 | 99 ± 10 | 102 ± 10 | No | 0.614638 | 99 | 102 | -3 | 5.774 | 0.5196 | 10 | 0.982462 |
| 8 | 87 ± 23 | 79 ± 16 | No | 0.502124 | 87 | 79 | 8 | 11.44 | 0.6994 | 8.921 | 0.982462 |
| 9 | 76 ± 8 | 76 ± 9 | No | >0.999999 | 76 | 76 | 0 | 4.916 | 0 | 9.864 | >0.999999 |
| 10 | 76 ± 19 | 58 ± 6 | No | 0.068966 | 76 | 58 | 18 | 8.134 | 2.213 | 5.987 | 0.54436 |
| 11 | 90 ± 21 | 68 ± 17 | No | 0.075299 | 90 | 68 | 22 | 11.03 | 1.995 | 9.584 | 0.54436 |
| n= 6 in each experiment | |  |  |  |  |  |  |  |  |  |  |

**Table S4**- xL3s motility in the medium and DMSO control groups, at 72 h of incubation. Statistical analysis and study of the existence of significant differences between the medium control group and the DMSO control group for each experiment.

| Incubation time | 72 h | | Unpaired t test with Welch correction and a multiple comparison Holm-Šídák post test, with a *P* < 0.05 | | | | | | | | |
| --- | --- | --- | --- | --- | --- | --- | --- | --- | --- | --- | --- |
| Experiment | Motility of medium control (mean ± SD) | Motility of DMSO control (mean ± SD) | Below threshold? | P value | Mean of medium control | Mean of DMSO control | Difference | SE of difference | t ratio | df | Adjusted P Value |
| 1 | 45 ± 15 | 52 ± 13 | No | 0.408325 | 45 | 52 | -7 | 8.103 | 0.8638 | 9.802 | 0.974615 |
| 2 | 50 ± 17 | 50 ± 14 | No | >0.999999 | 50 | 50 | 0 | 8.991 | 0 | 9.645 | >0.999999 |
| 3 | 76 ± 36 | 87 ± 15 | No | 0.512905 | 76 | 87 | -11 | 15.92 | 0.6909 | 6.685 | 0.974615 |
| 4 | 85 ± 18 | 97 ± 16 | No | 0.250642 | 85 | 97 | -12 | 9.832 | 1.221 | 9.864 | 0.922633 |
| 5 | 100 ± 26 | 90 ± 20 | No | 0.473516 | 100 | 90 | 10 | 13.39 | 0.7467 | 9.383 | 0.974615 |
| 6 | 90 ± 15 | 90 ± 19 | No | >0.999999 | 90 | 90 | 0 | 9.883 | 0 | 9.489 | >0.999999 |
| 7 | 53 ± 16 | 55 ± 11 | No | 0.806555 | 53 | 55 | -2 | 7.927 | 0.2523 | 8.863 | 0.992761 |
| 8 | 56 ± 22 | 69 ± 13 | No | 0.2475 | 56 | 69 | -13 | 10.43 | 1.246 | 8.112 | 0.922633 |
| 9 | 64 ± 12 | 54 ± 9 | No | 0.135909 | 64 | 54 | 10 | 6.124 | 1.633 | 9.273 | 0.767944 |
| 10 | 77 ± 25 | 49 ± 25 | No | 0.081095 | 77 | 49 | 28 | 14.43 | 1.94 | 10 | 0.605563 |
| 11 | 54 ± 10 | 58 ± 7 | No | 0.442949 | 54 | 58 | -4 | 4.983 | 0.8027 | 8.951 | 0.974615 |
| n= 6 in each experiment | |  |  |  |  |  |  |  |  |  |  |

**Table S5**- xL3s % of development in the medium and DMSO control groups, at 7 days of incubation. Statistical analysis and study of the existence of significant differences between the medium control group and the DMSO control group for each experiment.

| Incubation time | 7 days | | Unpaired t test with Welch correction and a multiple comparison Holm-Šídák post test, with a *P* < 0.05 | | | | | | | | |
| --- | --- | --- | --- | --- | --- | --- | --- | --- | --- | --- | --- |
| Experiment | % of development of medium control (mean ± SD) | % of development of DMSO control (mean ± SD) | Below threshold? | P value | Mean of medium control | Mean of DMSO control | Difference | SE of difference | t ratio | df | Adjusted P Value |
| 1 | 81.1 ± 8.2 | 74.0 ± 5.2 | No | 0.28547 | 81.1 | 74 | 7.1 | 5.606 | 1.267 | 3.385 | 0.813747 |
| 2 | 79.4 ± 8.9 | 75.3 ± 8.7 | No | 0.598821 | 79.4 | 75.3 | 4.1 | 7.186 | 0.5706 | 3.998 | 0.935433 |
| 3 | 77.9 ± 4.1 | 66.8 ± 5.8 | No | 0.060184 | 77.9 | 66.8 | 11.1 | 4.101 | 2.707 | 3.599 | 0.391387 |
| 4 | 84.1 ± 7.0 | 72.7 ± 3.9 | No | 0.086939 | 84.1 | 72.7 | 11.4 | 4.626 | 2.464 | 3.133 | 0.432162 |
| 5 | 80.0 ± 4.7 | 71.8 ± 2.0 | No | 0.077664 | 80 | 71.8 | 8.2 | 2.949 | 2.781 | 2.701 | 0.432162 |
| 6 | 66.5 ± 2.5 | 69.8 ± 4.0 | No | 0.303977 | 66.5 | 69.8 | -3.3 | 2.723 | 1.212 | 3.356 | 0.813747 |
| 7 | 80.5 ± 7.2 | 80.7 ± 3.3 | No | 0.968039 | 80.5 | 80.7 | -0.2 | 4.573 | 0.04374 | 2.805 | 0.968039 |
| 8 | 71.9 ± 5.1 | 70.8 ± 2.1 | No | 0.75525 | 71.9 | 70.8 | 1.1 | 3.184 | 0.3454 | 2.659 | 0.940097 |
| n= 3 in each experiment | |  |  |  |  |  |  |  |  |  |  |

**Table S6-** Commercial anthelmintic drugs and their activity at different concentrations for each *Haemonchus contortus* assay. Bold numbers indicate that the drug was active at the concentration tested.

|  |  |  | | |
| --- | --- | --- | --- | --- |
| Drug concentration (µM) | | % of motility on xL3s average (%CV)^a^ | % of L4 development average (%CV)^b^ | Adult stage motility score average (%CV)^a^ |
| Ivermectin | 1 | **25.8 (77.9)^c^** | **1.4 (334.0)^d^** | **0.7 (123.7)^e^** |
|  | 0.1 | 106.0 (35.5) | **37.0 (44.5)^d^** | **0.6 (57.7)^e^** |
|  | 0.01 | 104.9 (38.8) | 66.9 (53.6) | **0.7 (99.0)^e^** |
|  | 0.001 | NT | 86.7 (42.8) | **0.8 (65.0)^e^** |
|  | 0.0001 | NT | NT | **1.3 (13.3)^e^** |
| Levamisole | 50 | **0.6 (461.9)^c^** | **0.0 (NC)^d^** | **NT** |
|  | 5 | 72.6 (32.0) | **8.9 (72.0)^d^** | **0.0 (NC)^e^** |
|  | 0.5 | 128.1 (34.6) | 89.5 (6.0) | **0.0 (NC)^e^** |
|  | 0.005 | NT | NT | 1.9 (18.2) |
|  | 0.0005 | NT | NT | 2.8 (6.2) |
| Monepantel | 2 | **3.5 (183.1)^c^** | **4.0 (47.6)^d^** | **0.3 (115.5)^e^** |
|  | 0.2 | **9.3 (132.2)^c^** | **20.1 (155.1)^d^** | **0.7 (49.5)^e^** |
|  | 0.02 | 127.2 (35.8) | 85.5 (41.5) | **1.0 (34.6)^e^** |
|  | 0.002 | NT | NT | 2.2 (7.9) |
| Albendazole sulfoxide | 36 | **37.5 (67.0)^c^** | **0.0 (NC)^d^** | **1.0 (0.0)^e^** |
|  | 3.6 | **56.4 (41.5)^c^** | **1.9 (191.4)^d^** | **1.4 (37.1)^e^** |
|  | 0.36 | 134.8 (35.3) | **55.1 (53.1)^d^** | **1.3 (26.6)^e^** |
|  | 0.036 | 116.2 (30.1) | 74.5 (35.8) | 2.3 (0.0) |

^a^ Measured at 72 h of drug exposure

^b^ Measured at 7 days of drug exposure

NT: not tested; NC: it was not possible to calculate it because the value of the mean is zero.

^c^ Motility was significantly different from motility of DMSO control (*P* < 0.05) (see *Statistical analysis* section for further details)

^d^ Development was significantly different from development of DMSO control (*P* < 0.05) ) (see *Statistical analysis* section for further details)

^e^ Motility score of a compound at 72 h time point ≤1.5 is considered active [[19](#_ENREF_19)]

Table S7- Activity of a novel series of benzimidazole derivatives (at 25 µM) for each *Haemonchus contortus* assay (incubation time of 72h for xL3s or adult stage motility test, and 7 days for L4 development test). Bold numbers indicate that the compound was considered active.

| Compound ID^a^ | % of motility on xL3s average (%CV) | % of L4 development average (%CV) | Adult stage motility score average (%CV) |
| --- | --- | --- | --- |
| 1a | 93.1 (52.1) | 67.2 (12.6) | 2.2 (31.5) |
| 1b | 131 (44.3) | 103.8 (16.0) | **1.5 (57.7)^d^** |
| 1c | 144.8 (47.2) | 106.1 (15.3) | 2.0 (0.0) |
| 1d | 177.6 (54.7)^b^ | 68.2 (46.7) | **0.1 (173.2)^d^** |
| 1e | 179.6 (48.1)^b^ | **30.9 (84.6)^c^** | **0.1 (173.2)^d^** |
| 1f | 113.8 (39.1) | 101.9 (9.2) | 2.0 (0.0) |
| 2a | 193.1 (45.9)^b^ | 111.2 (14.2) | **1.2 (158.8)^d^** |
| 2b | 131.0 (46.1) | 108.9 (10.7) | 2.3 (37.7) |
| 2c | 134.5 (43.7) | 101.4 (23.2) | **1.4 (61.9)^d^** |
| 3a | 193.3 (28.9^b^ | 105.5 (12.1) | 2.2 (31.5) |
| 3b | 156.7 (20.0)^b^ | 100.0 (14.4) | 2.2 (31.5) |
| ABZ ^e^ | **29.4 (77.2)^b^** | **0.6 (346.4)^c^** | **1.0 (52.0)^d^** |

^a^ The chemical synthesis and structure of the compounds was previously reported by [[24](#_ENREF_24)].

^b^ Motility was significantly different from motility of DMSO control (*P* < 0.05) (see *Statistical analysis* section for further details)

^c^ Development was significantly different from development of DMSO control (*P* < 0.05) ) (see *Statistical analysis* section for further details)

^d^ Motility score of a compound at 72 h time point ≤1.5 is considered active [[19](#_ENREF_19)]

^e^ ABZ was evaluated at a concentration of 20 µM.

**
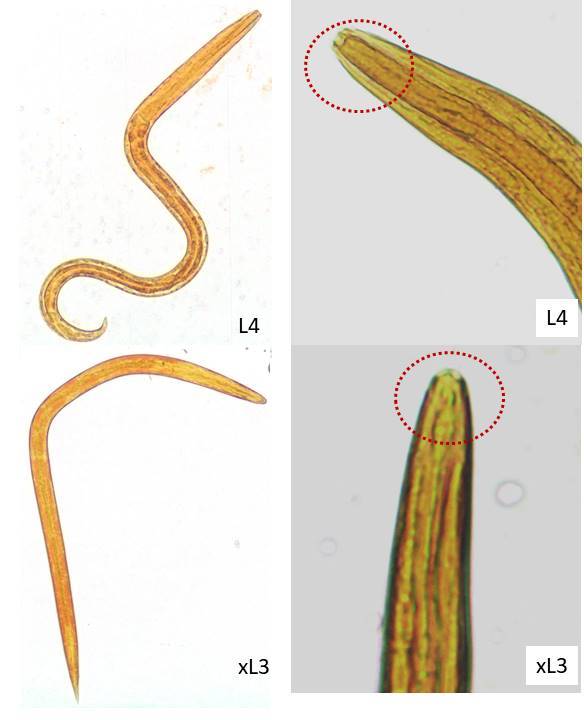
**

Figure S1- The present figure shows the morphological differences between xL3s and L4 stages of *Haemonchus contortus*, where the most notable distinction is the well-developed pharynx and the presence of a complete buccal capsule in L4 stage.


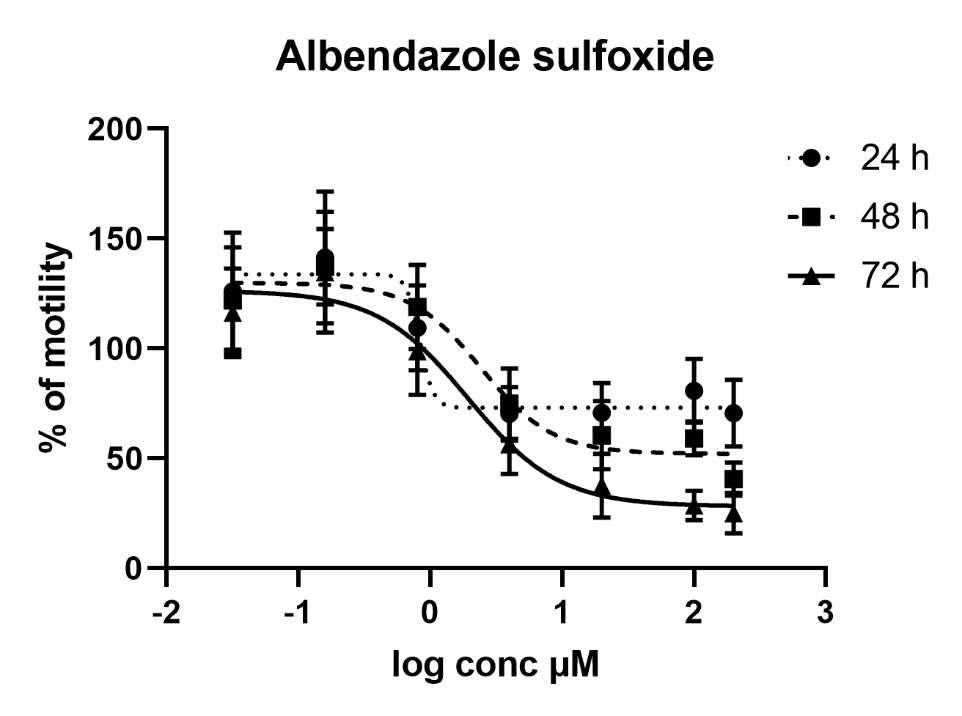


Figure S2- Albendazole sulfoxide dose- response curve in xL3s automated motility assay, at 24 h (dotted line), 48 h (dashed line) and 72 h (solid line). Each data point represents mean ± SEM, of three different experiments, with six replicates for each concentration.


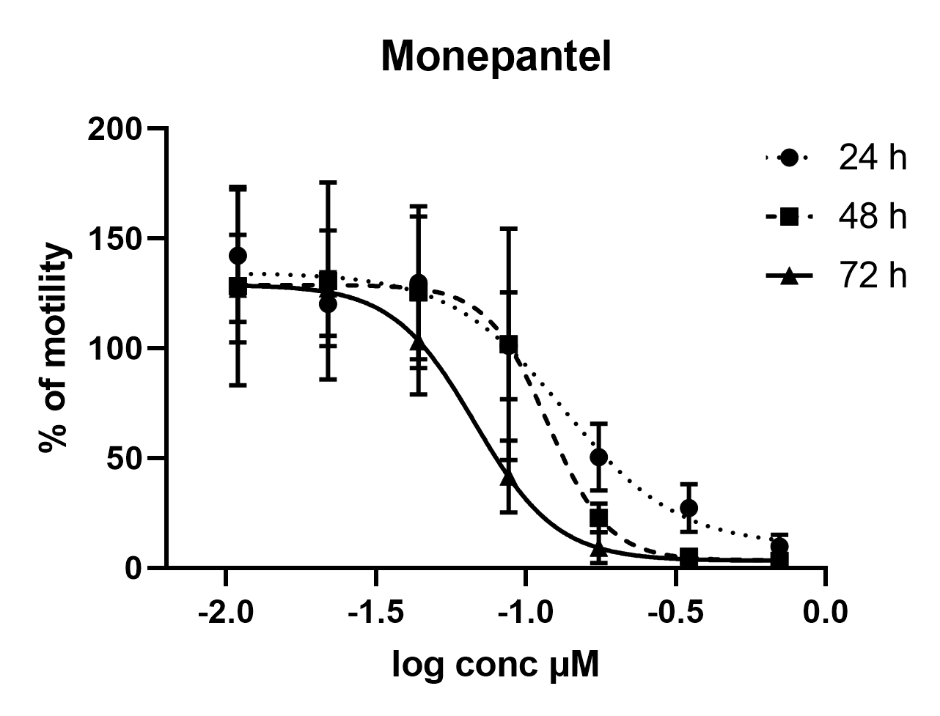


Figure S3- Monepantel dose- response curve in xL3s automated motility assay, at 24 h (dotted line), 48 h (dashed line) and 72 h (solid line). Each data point represents mean ± SEM, of three different experiments, with six replicates for each concentration.


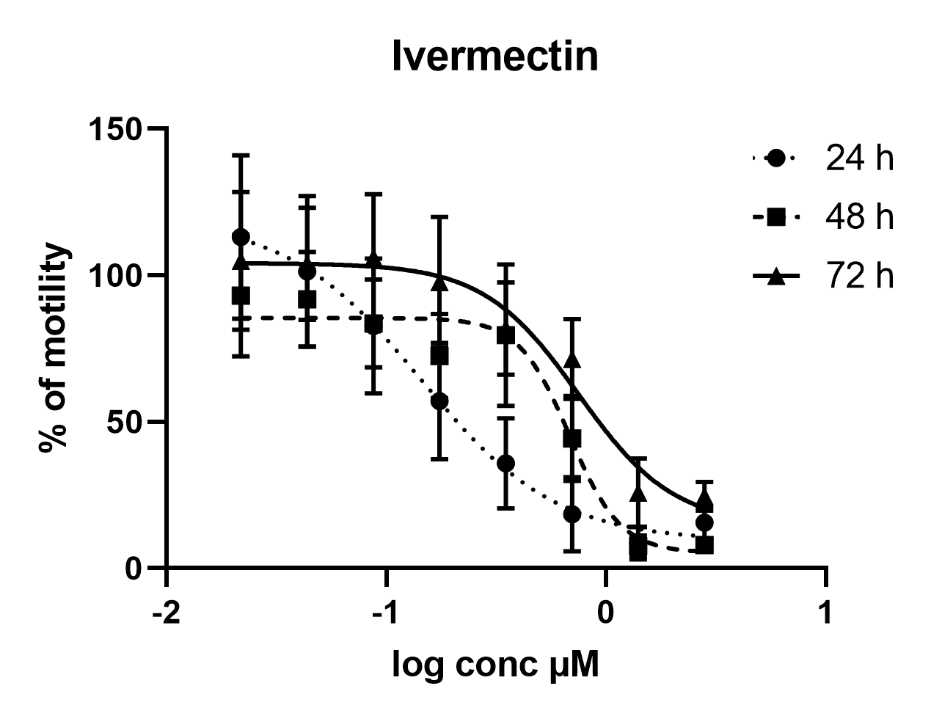


Figure S4- Ivermectin dose- response curve in xL3s automated motility assay, at 24 h (dotted line), 48 h (dashed line) and 72 h (solid line). Each data point represents mean ± SEM, of three different experiments, with six replicates for each concentration


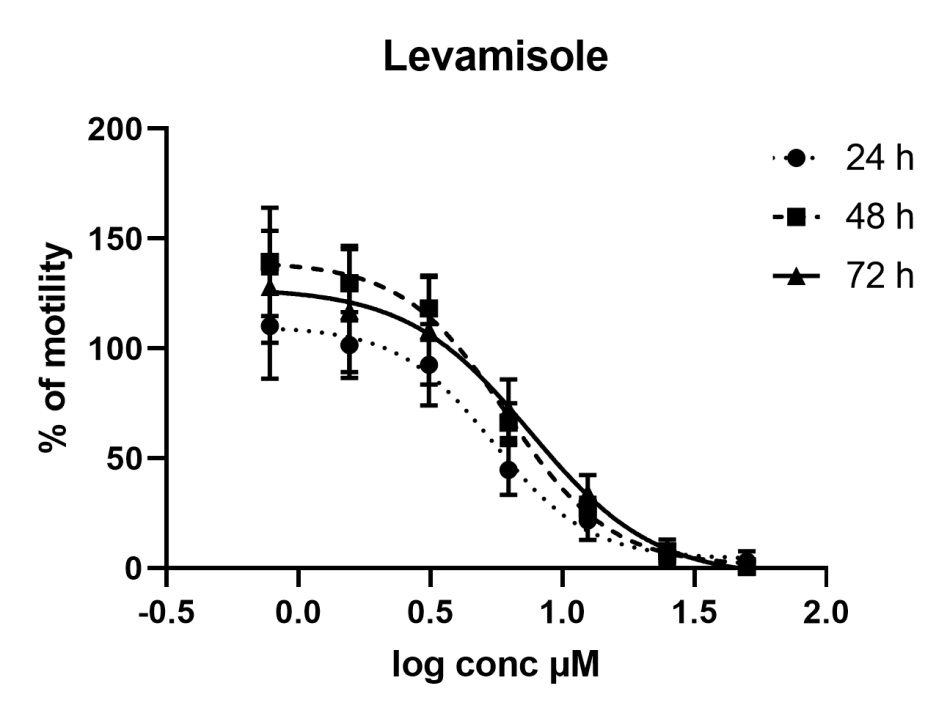


Figure S5- Levamisole dose- response curve in xL3s automated motility assay, at 24 h (dotted line), 48 h (dashed line) and 72 h (solid line). Each data point represents mean ± SEM, of three different experiments, with six replicates for each concentration


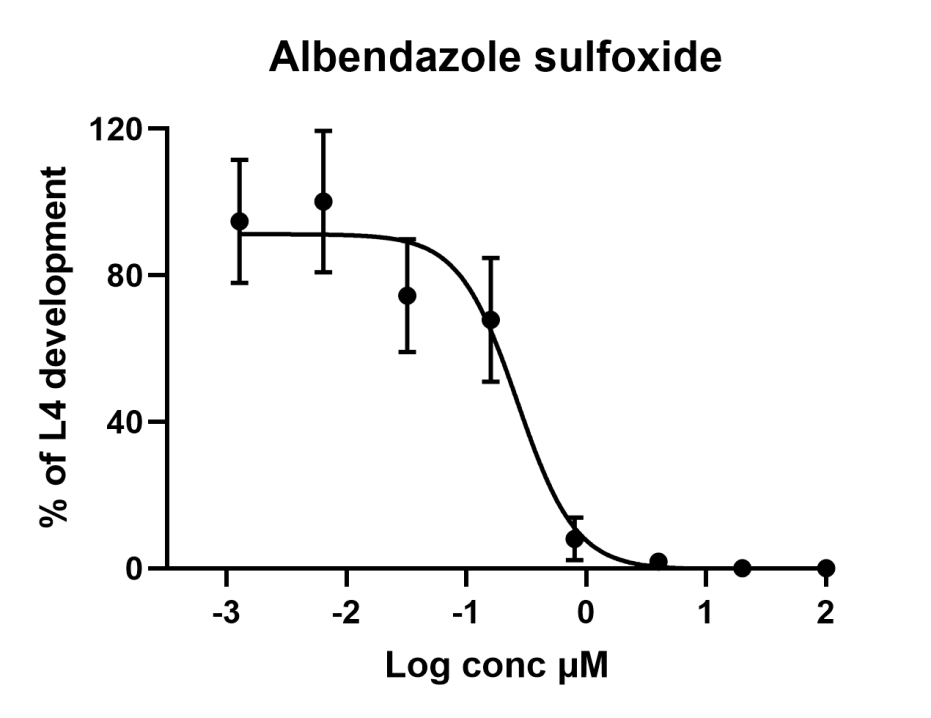


Figure S6- Albendazole sulfoxide dose- response curve in xL3s to L4 development assay. Each data point represents mean ± SEM, of three different experiments, with three replicates for each concentration


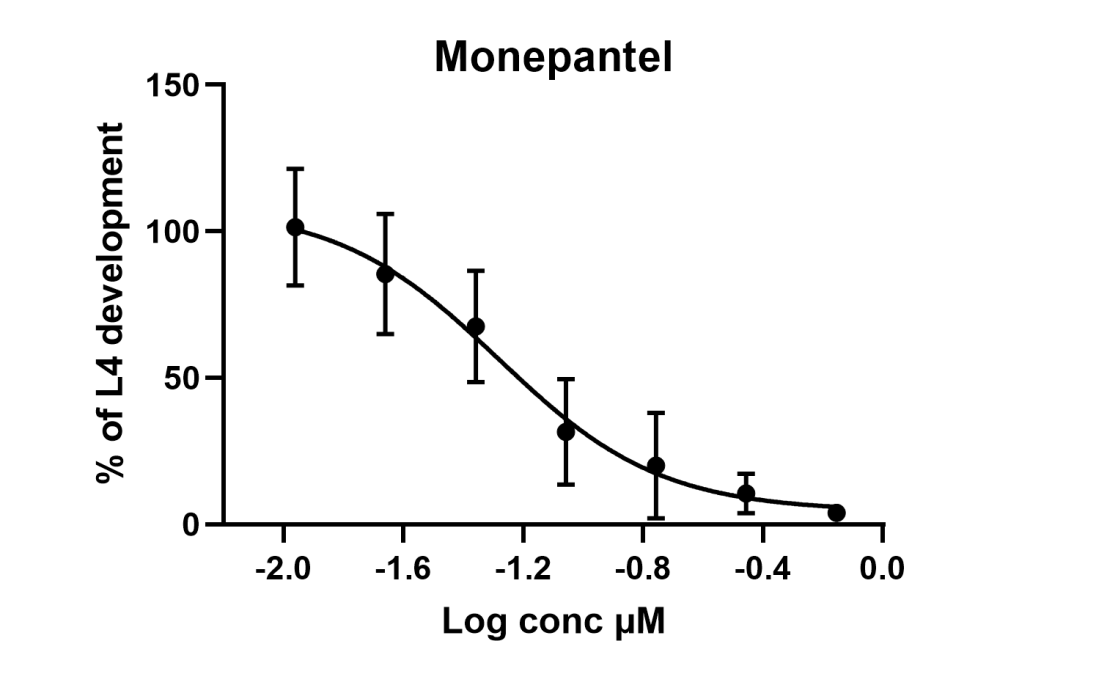


Figure S7- Monepantel dose- response curve in xL3s to L4 development assay. Each data point represents mean ± SEM, of three different experiments, with three replicates for each concentration


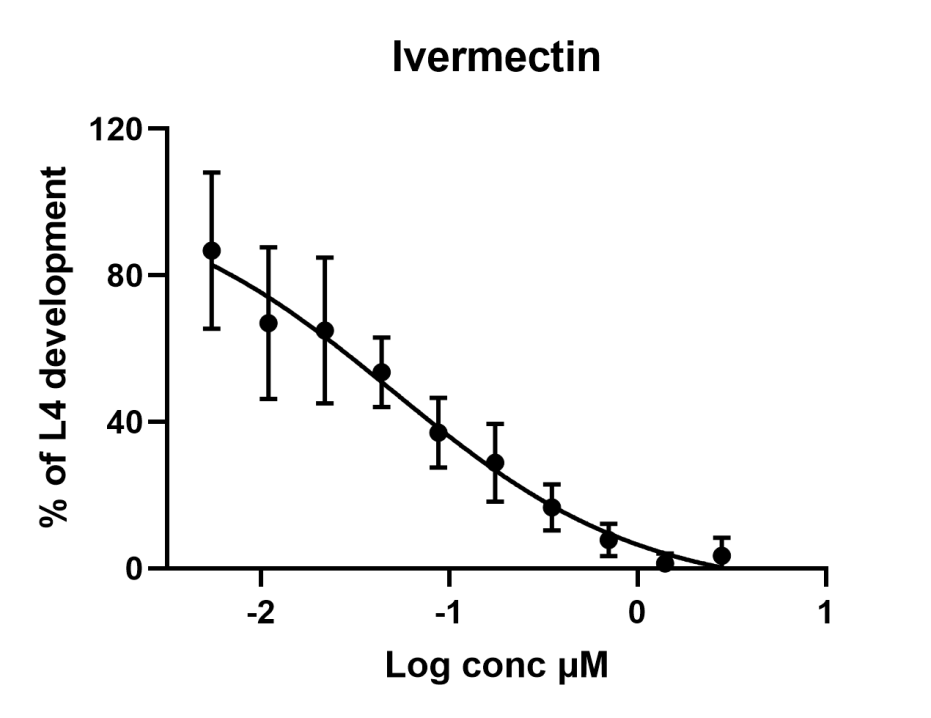


Figure S8- Ivermectin dose- response curve in xL3s to L4 development assay. Each data point represents mean ± SEM, of three different experiments, with three replicates for each concentration


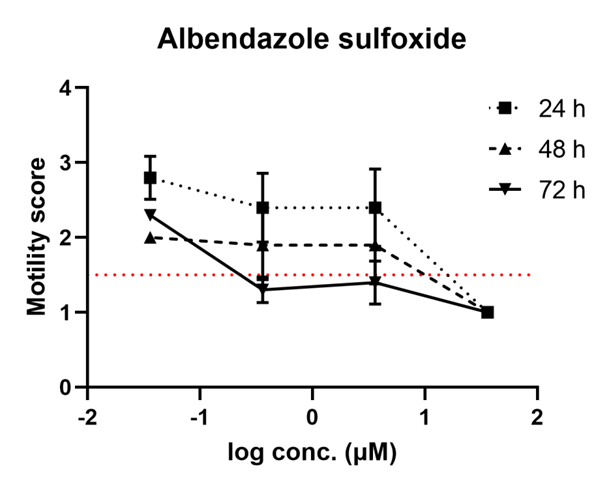


Figure S9- Albendazole sulfoxide effect on motility of *Haemonchus contortus* adult stage, at 24 h (dotted line), 48 h (dashed line) and 72 h (solid line). Each data point represents mean ± SEM motility score, of three different experiments, with six replicates for each concentration. Red dotted line indicates a motility score of 1.5, value to which a product is considered active.


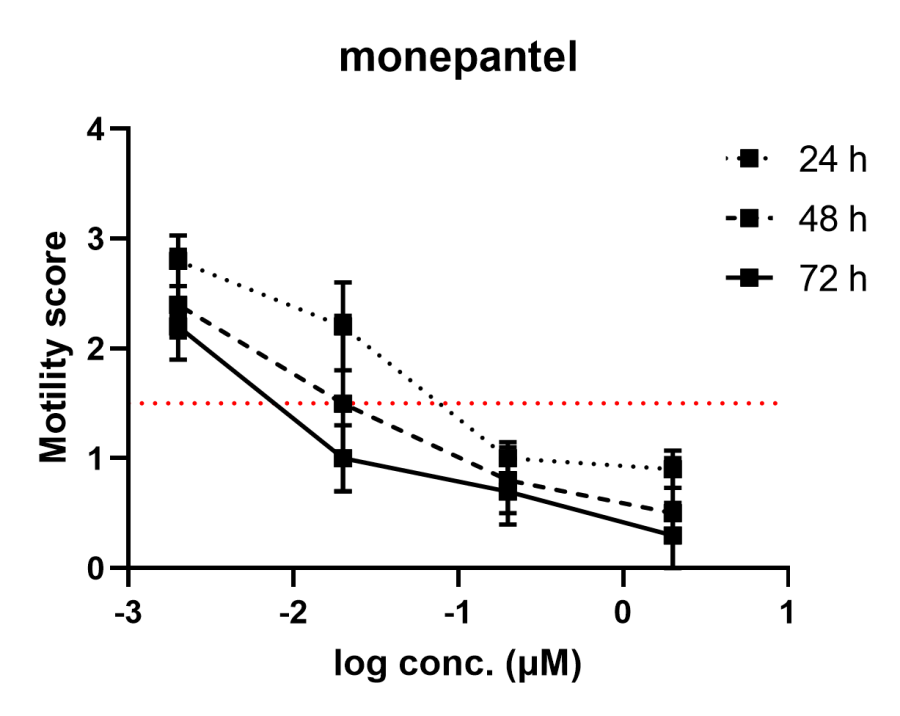


Figure S10- Monepantel effect on motility of *Haemonchus contortus* adult stage, at 24 h (dotted line), 48 h (dashed line) and 72 h (solid line). Each data point represents mean ± SEM motility score, of three different experiments, with six replicates for each concentration. Red dotted line indicates a motility score of 1.5, value to which a product is considered active.


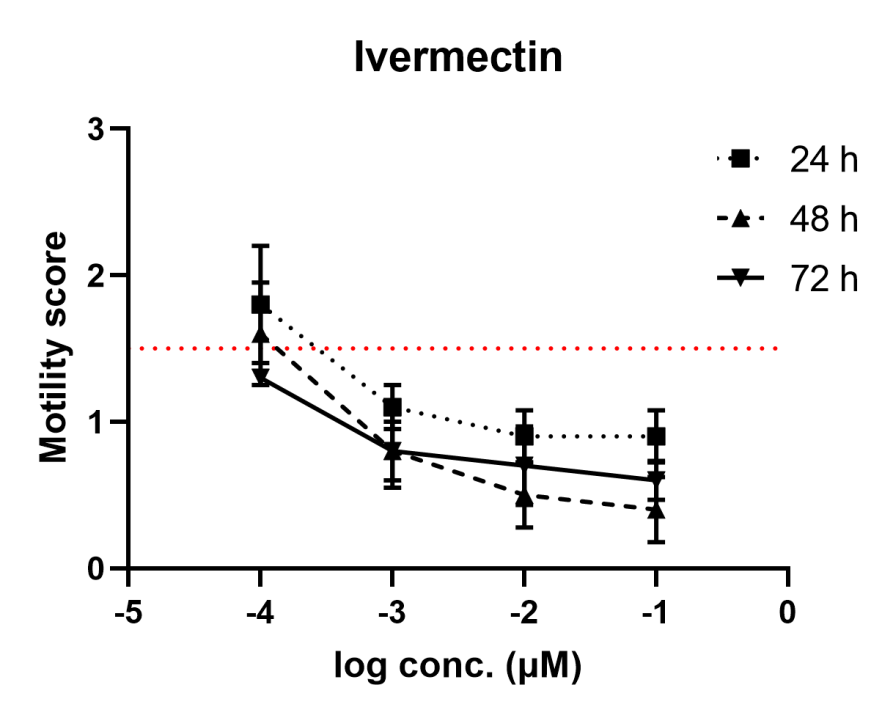


Figure S11- Ivermectin effect on motility of *Haemonchus contortus* adult stage, at 24 h (dotted line), 48 h (dashed line) and 72 h (solid line). Each data point represents mean ± SEM motility score, of three different experiments, with six replicates for each concentration. Red dotted line indicates a motility score of 1.5, value to which a product is considered active.


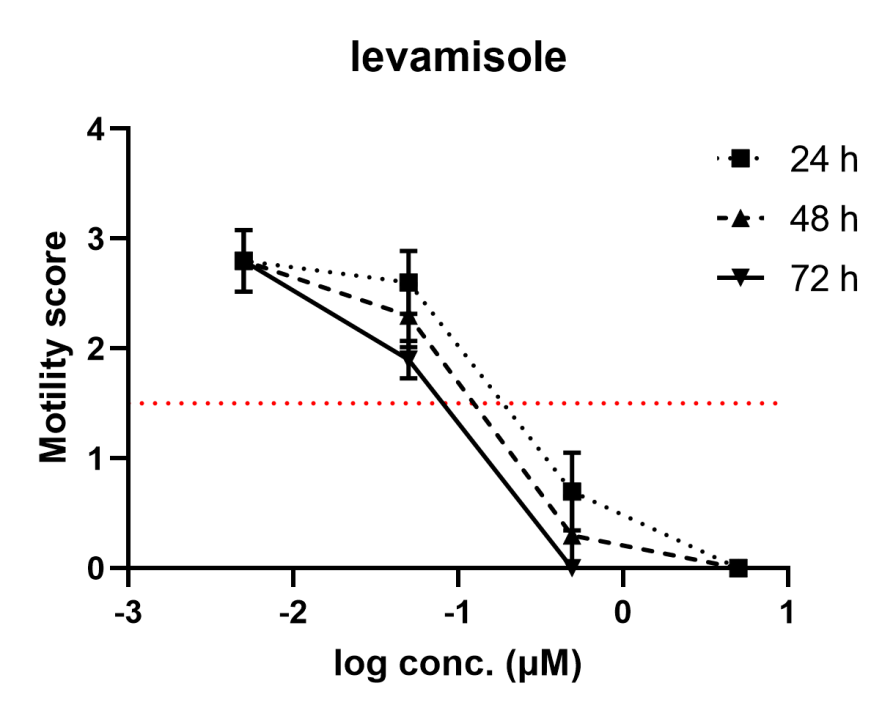


Figure S12- Levamisole effect on motility of *Haemonchus contortus* adult stage, at 24 h (dotted line), 48 h (dashed line) and 72 h (solid line). Each data point represents mean ± SEM motility score, of three different experiments, with six replicates for each concentration. Red dotted line indicates a motility score of 1.5, value to which a product is considered active.
